# Enhanced Recovery Pathway in Lung Resection Surgery: Program Establishment and Results of a Cohort Study Encompassing 1243 Consecutive Patients

**DOI:** 10.3390/cancers14071745

**Published:** 2022-03-29

**Authors:** Yen-Lan Nguyen, Elena Maiolino, Vincent De Pauw, Mathilde Prieto, Antonio Mazzella, Jean-Baptiste Peretout, Agnès Dechartres, Christophe Baillard, Antonio Bobbio, Elisa Daffré, Marco Alifano

**Affiliations:** 1 Anesthesiology and Critical Care Medicine Department, Cochin Academic Hospital, APHP, Université de Paris, 75014 Paris, France; yen-lan.nguyen@aphp.fr (Y.-L.N.); jean-baptiste.peretout@aphp.fr (J.-B.P.); christophe.baillard@aphp.fr (C.B.); 2 Thoracic Surgical Department, Cochin Academic Hospital, APHP, Université de Paris, 75014 Paris, France; elena.maiolino@aphp.fr (E.M.); vincent.depauw@aphp.fr (V.D.P.); mathilde.prieto@aphp.fr (M.P.); antonio.mazzella86@gmail.com (A.M.); antonio.bobbio@aphp.fr (A.B.); elisa.daffre@aphp.fr (E.D.); 3 Département Biostatistique Santé Publique et Information Médicale, INSERM, Institut Pierre Louis d’Epidémiologie et de Santé Publique, Sorbonne Université, Hôpitaux Universitaires Pitié Salpêtrière-Charles Foix, AP-HP, 75013 Paris, France; agnes.dechartres@aphp.fr

**Keywords:** enhanced recovery pathway, lung surgery, chest tube management

## Abstract

**Simple Summary:**

Enhanced Recovery Pathways (ERP) have been scarcely assessed in lung cancer surgery. We performed a two-step audit for our experience: the first dealing with our initial experience focusing on patients undergoing segmentectomies and lobectomies, the second including all subsequent consecutive patients undergoing all kind of lung resections for NSCLC. The first step aimed at auditing results of ERP on occurrence of postoperative complications and at assessing which ERP components were associated with improved short-term outcomes. We also audited late results by assessing long-term survival of patients in the first step of our study. The second step aimed at auditing on large-scale short-term results of the ERP in a real-life setting. In total, 166 patients were included in the first period. No postoperative death occurred. The overall adverse events rate was 30%. In multivariate analyzes, the only element associated with reduced adverse postoperative events was chest tube withdrawal within POD2. The 1-, 3- and 5-year survival rates were 97%, 86.1%, and 76.3%, respectively. In the second period, 1077 patients were included; 11 patients died during the postoperative period. The overall postoperative adverse event rate was 30.3%. Thoracoscore independently predicted postoperative death, the occurrence of complications (all-kind, minor, major, or respiratory ones). We conclude that compliance to ERP procedures and early chest tube removal are associated with reduced postoperative events in patients with lung resection surgery. Thoracoscore is a useful tool in predicting mortality and postoperative adverse events.

**Abstract:**

Introduction: In spite of increasing diffusion, Enhanced Recovery Pathways (ERP) have been scarcely assessed in large scale programs of lung cancer surgery. The aim of this study was auditing our practice. Methods: A two-step audit program was established: the first dealing with our initial ERP experience in patients undergoing non-extended anatomical segmentectomies and lobectomies, the second including all consecutive patients undergoing all kind of lung resections for NSCLC. The first step aimed at auditing results of ERP on occurrence of postoperative complications and at assessing which ERP components are associated with improved short-term outcomes. We also audited late results by assessing long-term survival of patients in the first step of our study. The second step aimed at auditing on large-scale short-term results of the ERP in a real-life setting. Results: Over a one-year period, 166 patients were included. The median number of ERP procedures per patient was three (IQR 3–4). No postoperative death occurred. The overall adverse events rate was 30%. In multivariate analyzes, the only element associated with reduced adverse postoperative events was chest tube withdrawal within POD2 (OR = 0.21, 95% CI (0.10–0.46)). The 1-, 3-, and 5-year survival rates were 97%, 86.1%, and 76.3%, respectively. In the second period, 1077 patients were included in our ERP; 11 patients died during the postoperative period or within 30 days of operation (1.02%). The overall postoperative adverse event rate was 30.3%, major complication occurring in 134 (12.4%), and minor ones in 192 (17.8%). Respiratory complications occurred in 64 (5.9%). Thoracoscore independently predicted postoperative death, the occurrence of complications (all-kind, minor, major, or respiratory ones). Conclusions: Compliance to ERP procedures and early chest tube removal are associated with reduced postoperative events in patients with lung resection surgery. In a large setting scale, ERP can be applied with satisfactory results in terms of mortality and morbidity. Thoracoscore is a useful tool in predicting mortality and postoperative adverse events.

## 1. Introduction

Lung resection remains the mainstay of treatment of localized and of a subset of locally advanced lung cancers. Despite technical progresses in surgery and anesthesiology, around 30% to 40% of patients experience postoperative complications leading to prolonged hospital stays and impaired quality of life [1]. This has an impact on long-term overall and disease-free survival, with occurrence of complications being an independent predictor of worse long-term outcome [2].

Despite the lack of recommendations, several teams have developed during the last 20 years clinical programs of enhanced recovery pathways (ERPs) in elective lung resection [3,4]. ERP is a standardized interdisciplinary peri-operative care program which aims to empower the patient, minimize surgical stress, improve functional recovery, and reduce postoperative complications. A recent systematic review on these programs suggests that they might be associated with reduced length of hospital stay and costs [5].

Recently, a consensual ERP after elective lung resection was implemented in our department. This ERP program included optimization of patient empowerment (through dedicated information leaflets), analgesia (with patient controlled loco-regional anesthesia and morphine sparing), nutrition (with pre-operative carbohydrates consumption, diminution of preoperative fasting and early postoperative re-nutrition), chest physiotherapy (early mobilization), and chest tube management (use of a single chest tube and early removal). 

Immediately after initial development of the program, we decided to prospectively study short and long-term outcome of enrolled patients. A two-step audit program was established: the first (one year) dealing with patients undergoing anatomical non-extended lung resection (segmentectomy and lobectomy), the second (two years) including all consecutive patients undergoing surgery (including extended lobectomy and pneumonectomy) for NSCLC. The first step of this study aim at auditing results of ERP on occurrence of postoperative complications and at assessing which ERP components are associated with improved short-term outcomes. We also audited late results of our operative management of lung cancer, by assessing long-term survival of patients in the first step of our study. The second step aim at auditing on large-scale short-term results of the ERP in a real-life unselected population of patients with resectable lung cancer referred to a single high-volume surgical center. The idea of starting our ERP program with patients undergoing either standard lobectomy or segmentectomy came from the consideration that this would facilitate the implementation of the new medical and nursing procedures (due to difficulties in changing clinical practices). The benefits observed (audits of ERP compliance and outcomes were regularly made) and the adhesion of all health care providers (surgeons, anesthesiologists, intensivist, nursing staff, nurses, helpers, physiotherapists, dietitians), lead us to broaden our selection criteria, and to implement ERP to all patients undergoing lung resections in the second period

## 2. Patients and Methods

### 2.1. Study Design

This is a single-center observational cohort study. Ethical approval for this study was obtained by the Institutional Review Board “Comité de Protection des Personnes CPP Ile de France III” (file 2015-A01566-43). Informed consent was obtained by all patients.

### 2.2. Setting and Timing

Cochin hospital is a large tertiary referral academic center. Our ERP started in October 2015. Two subsequent periods were analyzed in this study. The first period (October 2015–November 2016) deals with consecutive patients scheduled for anatomical non-extended segmentectomy or lobectomy for proven or suspected NSCLC managed according to ERP protocol established during the phase of setting. At the same time, all the remaining patients underwent perioperative management according to the pre-ERP standards. At the completion of the first period we performed during the month of December 2016 an internal audit of initial results (Nguyen, Meetings if French Society of Anesthesiology and Critical Care Medicine (SFAR 2017) and of the European Society of Intensive Care Medicine (2018)) leading to the decision of adopting the ERP protocol to all subsequent patients undergoing lung surgery for resectable lung cancer, regardless of clinical staging and anticipated extent of resection. On January 2017, we restarted prospective collection of data on patients with proven or suspected non-small cell lung cancer undergoing surgery. At this time, we started prospective collection of a further parameter, thoracoscore [6], a score of surgical risk developed by the French Society of Thoracic and Cardiovascular surgery, taking into account nine patient-related parameters, namely age, sex, ASA classification, WHO performance status classification, dyspnea score, priority of surgery (urgent or not), contamination class of surgery, comorbidity score, and diagnosis group (benign or malignant). Thus, the second period of the study deals with our first unselected population of 1077 patients managed according to the ERP principles. 

### 2.3. Enhanced Recovery Program

#### 2.3.1. Pre-Operative Assessment

For every patient, preoperative evaluation consisted of history taking and physical examination, routine blood tests, and pulmonary function tests. Cardio-respiratory risk assessment was performed according to American College of Chest Physicians guidelines. All patients underwent routine oncologic preoperative work-up including fiberoptic bronchoscopy, thoracic, and upper abdominal contrast-enhanced CT scan, together with cerebral CT scan or Magnetic Resonance Imaging (MRI). Then, 18FDG CT coupled positron emission tomography was also routinely employed. Invasive mediastinal staging (endobronchial ultrasound-EBUS or mediastinoscopy/video-assisted thoracoscopy) was indicated in case of enlarged (short-axis > 1 cm) and/or hypermetabolic lymph nodes. Patients with proven N2 disease underwent platinum-based neoadjuvant chemotherapy followed by surgery in case of response or stable diseases. Adjuvant treatment was proposed on an individual basis under the care of referring pneumologist or oncologist, following multidisciplinary guidelines and evidence-based discussions. 

#### 2.3.2. Pre-Operative Pathway

We distributed to ERP patients specifically dedicated information leaflet including detailed explanations of the intended perioperative pathway (importance of smoking cessation, proposal of specialized consultation or nicotine supplements), exercise training before surgery, nutritional support, shorter preoperative fasting, pain control, early respiratory and motor rehabilitation. Patients were instructed on the importance of their active role in the peri-operative management, particularly with respect to pain management and early mobilization. All patients were admitted to hospital on the day before surgery. They were given carbohydrates loading (NUTRICIA PreOp^®^) in the evening prior surgery and two hours before induction of anesthesia, to prevent dehydration and reduce postoperative insulin resistance and anxiety (Table 1). 

#### 2.3.3. Per-Operative Pathway

All patients had air insulation blankets and warmed fluids to avoid hypothermia. General anesthesia was performed with targeted-controlled propofol infusion with bispectral index monitoring and targeted-controlled sufentanil infusion. Muscle relaxation was conducted with atracurium (0.5 mg/Kg) and monitored with train-of-four stimulation at ulnar nerve. Ventilation was performed with a double lumen endotracheal tube. During two-lung ventilation, volume-controlled mode settings included a tidal volume between 6 to 8 mL/Kg ideal body weight and a positive end-expiratory pressure between 5 to 8 cm H_2_O. During one-lung-ventilation, volume-controlled mode settings encompassed a tidal volume of 4–5 mL/Kg ideal body weight and a positive end-expiratory pressure 3–6 cm H_2_O. In both cases, alveolar recruitment maneuvers were conducted at the discretion of the anesthesiologist. 

All patients had limited volume expansion, prevention of nausea and vomiting with 8 mg dexamethasone, a protocol of systemic analgesics (paracetamol 1000 mg, nefopam 20 mg, and ketoprofene 50 mg) and 1.25 mg/mL of chirocaine distributes in a paravertebral anesthesia catheter.

Surgical approach was left at discretion of senior surgeon and consisted of limited postero-lateral thoracotomy, bi-portal or uni-portal video-assisted thoracoscopy. Lobectomy was the operation of choice; pneumonectomy was carried out when lobar resection (including bronchial and bronchovascular sleeve resections) was not possible. Sublobar resections were generally performed in case of poor respiratory reserve, multiple primaries or cT1a tumors. Anyway, with respect to infralobar resections, these rules were not strict and segmentectomy could be decided in the setting of a Multidisciplinary Team Meeting, taking into account patient fitness, operative risk, and biological age. Nodal dissection was systematically carried out, but special care was adopted to maximally preserve bronchial vascular supply. At the end of operation, general policy was the use of a single chest tube (24F). Patients were extubated in the operating room. 

#### 2.3.4. Postoperative Pathway

Patients were allowed to drink and to eat a light meal two and four hours, respectively, after extubation. All patients were mobilized within 12 h after surgery and had at least twice daily physiotherapist sessions. A chest tube protocol recommended the withdrawal of aspiration at POD 1 and chest tube removal on the morning of POD 2 if there was no air leak or bloody or chylous effusion. 

### 2.4. Statistical Analysis

For the first part of the study, we analyzed specifically 6 ERP procedures (new medical procedures):

- Smoking cessation;
- Carbohydrates intake;
- Personalized controlled regional anesthesia;
- Opiate analgesia avoidance strategy with non-steroid anti-inflammatory drugs use at day 0;
- Opiate analgesia avoidance strategy with non-steroid anti-inflammatory drugs use at day 1;
- Chest tube removal at day 2.

Descriptive analysis involved frequencies and percentages for qualitative variables and the mean (±SD) or the median (Q1–Q3) as appropriate for quantitative variables. 

Complications were prospectively collected and classed according to the Clavien and Dindo classification [7]. We identified factors associated with complications in both periods. For the first period, in univariate analysis, we compared general characteristics, type and extent of the surgical intervention and components of the ERP program between patients with and without complications using Chi square tests or Fishers’ exact tests as appropriate for qualitative variables and Student *t* test or Wilcoxon tests for quantitative variables. Then, we conducted a multivariate logistic regression model including all variables with a *p*-value < 0.05 in univariate analysis to identify factors independently associated with occurrence of complications. In the second period (after complete implementation of the ERP program), we used the same statistical approach to identify factors associated with the occurrence of postoperative death, minor or major complications and, particularly, respiratory complications.

#### Long-Term Postoperative Outcomes

Survival rate of the first period was assessed at 5 years using the local information system and the national mortality database of the Institute National de la statistique et des études économiques (INSEE), including all the deaths occurred in any French municipality. With respect to study of survival, Kaplan–Meier estimates were obtained and survival curves were compared by the log-rank test. Multivariate analysis was performed by the Cox model. All analyses were conducted with SAS version 9.3. A *p*-value < 0.05 was considered as statistically significant.

## 3. Results

### 3.1. First Period 

#### 3.1.1. Patient Population 

In total, 166 patients were included in our enhanced recovery pathway program between October 2015 and November 2016 (Table 2). Around half of them were men (*n* = 86; 51.8%); mean age was 66 ± 10 years and mean body max index was 25 ± 4.7 Kg/m^2^. The majority of patients had an American Society of Anesthesiologists (ASA) score at 2 (*n* = 142 (85.5%)). Less than one third of patients (*n* = 36) had sub-lobar resection. The others had lobectomy (*n* = 130; 78.3%); the most common surgical approach was thoracotomy (*n* = 149; 89.8%). 

Overall, 68% of the patients had one or more comorbidities: one third of patients had chronic obstructive pulmonary disease (*n* = 59) and 10.2% (*n* = 17) had coronary artery disease. Pre-operative chemotherapy was administrated in 6.6% of the cases; around one in 15 patients (*n* = 17) had a planned postoperative hospitalization in the intermediate care unit, whereas all the remaining patients went postoperatively in the thoracic surgery ward. 

#### 3.1.2. Enhanced Recovery Compliance 

The median number of ERP procedures per patient was 3 (IQR (3–4)). Two-thirds of smokers (*n* = 133; 66%) stopped smoking before surgery. During the pre-operative period, the majority of patients had oral carbohydrates loading before surgery (*n* = 123; 74%). 

Postoperatively, pain measured at mobilization was greater than pain measured at rest with maximal pain at mobilization of 6 ± 3 on day 0, 6 ± 2 on postoperative day (POD) 1 and 2, and maximal pain at rest of 5 ± 4 on day 0, 4 ± 2 on POD1 and 4 ± 3 on POD2. The median consumption of morphine was 3 ± 4 mg at day 0 and 0 ± 7 mg at POD1. 

Adverse postoperative events are shown in Table 3. The overall adverse events rate was 30% (*n* = 50), of which almost half 17% (*n* = 29) was respiratory. Patients hospitalized in the intermediate care unit (OR 7.26 (2.40–21.9)) and those with a chest tube that was kept beyond POD2 (OR 0.99 (0.51–1.94)) were more likely to have adverse postoperative events. In multivariate analysis, the only element associated with reduced adverse postoperative events was chest tube withdrawal within POD2 (OR 0.21 (0.10–0.46)), the remaining element not reaching significance (smoking cessation OR 0.50 (0.21–1.18); Carbohydrates intake OR 1.14 (0.46–2.84); patient controlled regional anesthesia OR 0.82 (0.41–1.61); non-steroids anti-inflammatory drugs at day 0 OR 0.54 (0.27–1.06); non-steroids anti-inflammatory drugs at day 1 OR = 0.46 (0.19–1.10).

The number of adopted ERP procedures was also associated with the occurrence of adverse postoperative events, with crude ORs of 0.10 (95% CI 0.009–1.10, *p* = 0.06), 0.12 (0.013–1.13, *p* = 0.06), 0.08 (0.008–0.76, *p* = 0.03), and 0.02 (0.0001–0.35, *p* = 0.008) when comparing 2, 3, 4, and 5 procedures versus 1, respectively. Adjusted (for age, COPD, and need of intermediate care unit hospitalization) ORs were 0.09 (0.008–1.11, *p* = 0.06), 0.11 (0.01–1.11, *p* = 0.06), 0.08 (0.008–0.87, *p* = 0.04), and 0.02 (0.0001–0.39, *p* = 0.01), respectively. 

The median postoperative hospital stay was 6 days [5,6,7,8], and 84% (*n* = 139) patients returned home. One patient died during the first period (0.05%). 

#### 3.1.3. Long Term Survival

The median survival of the first period population was 60 months. One, 3- and 5-year survival rates were 97%, 86.1%, and 76.3%, respectively. Patients over 70-years-old had lower overall survival (*p* = 0.0066; 67.9% (54.5–78.9) versus 80.3 (71.9–86.6) at 5 years). Histological type (*p* = 0.026), pT (*p* = 0.049) and pN (*p* = 0.000079) parameters, pleural invasion (*p* = 0.0026), as well as pathological stage (*p* = 0.00015) were also associated with survival at univariate analysis (Table 4). At multivariate analysis (Table 5), age > 70 years remained independently associated with worse survival rate (HR = 2.5 (1.31–4.78), *p* = 0.0066), together with pathological stage (*p* = 0.00015; stage I: Ref, stage II: HR 2.28 (1.56–3.32); stage III HR: 5.18 (2.43–11.06)).

Occurrence of all-kind and of respiratory complications (Table 6) was associated with higher age (*p* < 0.001 and *p* = 0.0079), male sex (both *p* < 0.001), lower FEV1 (both *p* < 0.001) or FVC (*p* < 0.001 and *p* = 0.0026), lower DLCO (*p* < 0.001 and *p* = 0.031), higher Thoracoscore (both *p* < 0.001), smoking status (*p* = 0.011 and *p* = 0.0063), higher ASA score (both *p* < 0.001), presence of COPD (both *p* < 0.001), and histologic type (both *p* < 0.001). History of ischemic heart disease was associated with occurrence of all kind complications (*p* = 0.011), but not of respiratory complications.

### 3.2. Second Period

#### 3.2.1. Patient Population

From January 2017 to august 2021, 1077 patients were included in our enhanced recovery program (Table 2); 57.2% of them were male (*n* = 616), mean age was 66 ± 10 years and mean body max index was 25 ± 4.6 Kg/m^2^. One third of patients had chronic obstructive pulmonary disease (*n* = 419; 38.9%). Major comorbidities (more than three) were observed in 591 patients (54.9%) with 241 (22.4%) of them being represented by cardiac diseases. ASA scores were I in 38% of patients (*n* = 410), II in 41.8% (*n* = 450), and >II in 20.2% (*n* = 217). Pre-operative chemotherapy was administrated in 6.8% (*n* = 73) of the cases. Overall, 59.1% of patients underwent lobectomy (*n* = 636) whereas only one third of them (*n* = 294) had sub-lobar resections. The most common surgical approach was thoracotomy (*n* = 903; 83.8%). In total, 20% of smokers (*n* = 249) stopped smoking before surgery. Overall, 15.8% of patients (*n* = 170) had a planned hospitalization in the intermediate care unit. 

#### 3.2.2. Early Outcomes

A total of 11 patients died during the postoperative period or within 30 days of operation (1.02%). The overall postoperative adverse event rate was 30.3% (*n* = 326); 17.8% (*n* = 192) were minor complications and 12.4% (*n* = 134) were major complications. Major complications were mainly represented by bleeding (*n* = 23, 17.2%), pneumonia (*n* = 45; 33.6%), and ARDS (*n* = 14, 10.4%) (Table 3). 

Table 6 shows results of univariate analysis of baseline clinical and pathological factors associated with early postoperative outcomes: in-hospital or 30-day mortality, occurrence of all-kind complications (no, minor, major), and occurrence of respiratory complications. We included in the analysis the extent of exeresis in the idea that need of pneumonectomy was determined by tumor bulk and location, thus being a baseline characteristic independent by surgical or perioperative management.

Death was associated with higher age (*p* = 0.031), male sex (*p* = 0.049), lower FEV1 (*p* = 0.0017) or FVC (*p* = 0.0020), higher Thoracoscore (*p* = 0.0024), and higher ASA score (*p* = 0.048). At multivariate analysis, death was independently associated only with thoracoscore > 2.3 (75° percentile) (*p* = 0.0065, OR 6.94 (1.72–28.02)).

Two models (Table 7) were built to assess parameters independently associated with the occurrence of complications (regardless of gravity) and of major complications only, respectively. Occurrence of all-kind of complications was independently associated with male sex (*p* = 0.00004, OR 1.95 (1.42–2.67)), age > 70 years (*p* = 0.025, OR 1.42 (1.04–1.93)), and thoracoscore > 2.3 (*p* = 0.00049, OR 1.85 (1.31–2.62)), with FEV1 > 80% of predicted being protective (*p* = 0.004, OR 0.63 (0.46–0.86)). Occurrence of major complications was independently associated with male sex (*p* = 0.0015, OR 2.19 (1.35–3.55)), thoracoscore > 2.3 (*p* = 0.00017, OR 2.30 (1.49–3.56)), and tobacco consumption (*p* = 0.043, no smoker = REF, former OR 1.43 (1.01–2.01), current OR 2.03 (1.02–4.05)).

A final model was built to assess factors independently associated with respiratory complications: male sex (*p* = 0.008, OR 2.90 (1.32–6.39)), thoracoscore > 2.3 (*p* = 0.000024, OR 3.63 (2.00–6.00)), and tobacco consumption (*p* = 0.0052, no smoker = Ref, former OR 2.12 (1.25–3.58), current OR 4.47 (1.57–12.79)) were independent predictors.

## 4. Discussion

### 4.1. Implementation of ERP Led to Changes in Clinical Practice

The implementation of ERP in our Department led to major changes particularly concerning information delivery, management of preoperative fasting, postoperative pain and chest tube policy. In particular, procedures such as the distribution of an information leaflet, the carbohydrates prescription, or the use of a single chest tube were promptly adopted. Indeed, prescribing non-steroid anti-inflammatory drugs required taking into account potential side effects, drugs interactions, but also emotional beliefs, such as “the fear of bleeding”. Furthermore, chest tube management remains often a “surgeon dependent” procedure based on “clinical experience” rather than evidence-based medicine [8]. An example of that is the systematic use of suction beyond POD1 on chest tubes despite the evidence of benefits [9]. The compliance rates to our procedures were similar to other studies [10,11]. Similarly to previous experiences, we found that increased compliance with ERP procedures was associated with good clinical outcomes, as outlined by low mortality rates [12,13]. Nonetheless, the number and nature of the elements included in ERP vary a lot across studies and recent data suggest that only a few are predictors of adverse postoperative events [12,14]. Then, considering the difficulties to change medical practice, it would be important in the future ERP recommendations, in order to help clinicians eager to implement an ERP pathway, to grade the different elements, not according to their evidence-based value studied alone, but within an ERP pathway.

### 4.2. Early Chest Tube Removal Is Associated with Reduced Adverse Postoperative Events

Similarly to the results of Madani et al. in their before-after ERP study, we found that among ERP elements, early chest tube removal was a key element influencing morbidity [14]. After thoracic surgery, having a chest tube is often associated with pain, discomfort, and shortness of breath with subsequent reduction in mobilization, particularly if suction is employed. Refai et al. found that early removal of chest tube reduces static and dynamic pain and improves ventilator function independently of surgical access [15]. Consequently, in the theoretical framework of the occurrence of postoperative pneumonia, a chest tube appears to be the core element, which may explain the association between early removal and morbidity. The two elements that limit early chest tube removal are the persistence of an ongoing air leak and the serous liquid volume production. The complexity of air leak measurement (presence of bubbles in water seal chamber following coughing) is due to the difficulty distinguishing between the remaining air being due to a pleural space effect or to an active air leak. Some authors suggest the use of digital chest drain system—not used in our practice—to enhance the evaluation of air leak severity and reduce inter-observers variability [16]. A meta-analysis suggests an association between the use of digital and shorter chest tube duration, but a recent randomized controlled trial did not show any benefit [17,18]. To date, there is no consensus on the maximal threshold of serous liquid volume allowing safe early chest tube removal, but the randomized controlled trial of Bjerregaardt et al. suggest that an output of up to 500 mL of serous fluid allows safe removal of chest tube without increased rate of need of pleurocentesis or reinsertion of chest tube [19]. Rogers and al. found that among ERP elements, carbohydrates intake and early deambulation were the only predictors of improved outcomes [12], but, interestingly, they did not take in account chest tube removal. So it is possible that early deambulation may be a surrogate marker of early chest removal. 

In comparison to the study of Van Haren et al., the median length of hospital stay observed in our initial sample size is much longer (4 ± 3 vs. 6 (5–8)) [20]. The surgical approach may not be the underlying reason. Indeed, Van Haren et al. did not find any length of hospital stay differences between patients benefiting from lung resection through minimally invasive surgery vs. thoracotomy [20]. Among underlying reasons, possibly the frequent long distances between our hospital and patients’ home might have influenced this parameter.

### 4.3. ERP and Long-Term Outcome

Lung cancer is highly lethal with a historical 5-year overall survival rate of approximately 60% in patients with localized disease undergoing surgery. In our study survival rates at 5 years seems satisfactory, with figures of 86.7%, 63.4% and 50% in pathological stage I, II, and III, respectively. Possibly, a relatively simple postoperative course contributed to good long-term survival, the occurrence of postoperative complications being an independent predictor of worse long-term outcome.

### 4.4. ERP Applied in a Large Scale Setting 

The second part of our study deals with a population of unselected patients undergoing resection for lung cancer. We confirmed that ERP can be applied in the setting of a large-scale program of general thoracic surgery, with results which seems fully satisfactory in terms of mortality and morbidity.

In the second part of our study we found that thoracoscore, a risk score in general thoracic surgery, was a useful tool in assessing operative risk of lung resection surgery and observed that thoracoscore > 2.3 (75° percentile) was independently associated with death and with the occurrence of complications, in particularly major and respiratory ones. Thoracoscore, was developed from EPITHOR, the official database of French Society of Thoracic and Cardiovascular Surgery which was created in 2002 and in the first publication by Falcoz et al. [6] its usefulness in predicting postoperative complications and death after all-kinds of general thoracic surgery procedures was proven [6]. This initial series dealt with patients operated on between 2002 and 2005 and postoperative mortality after lung surgery procedures was 2.5%. Our data show that Thoracoscore remains a valid and reliable clinical tool for predicting death and postoperative complications in thoracic surgery in the era of enhanced recovery pathways. Although not significant at multivariable analysis, the histologic type was associated with the occurrence of postoperative complications, which were more frequent in patients with epidermoid cancer, probably because of the known more important history of tobacco consumption and more frequent central location of the disease. 

### 4.5. Limitations

Our study had several limitations. As with any observational study in a single institution, it suffers from selection bias, unmeasured confounding factors, and external validity. Our Thoracic Surgery Department being a high-volume center with each surgeon practicing more than 100 lung resections per year, may be transposition of results might be difficult. Another particularity of our study is that the majority of patients had thoracotomy despite the modern tendency of minimally invasive surgery. Our local culture of such surgical approach is slowly evolving, but our data seems to suggest that other parameters than surgical approach are main determinants of both short and long term outcome.

## 5. Conclusions

In conclusion, The implementation of an ERP lead to major changes in our clinical practices. In our experiences patients undergoing lung resection within ERP had satisfactory short- and long-term results. Thoracoscore, a predictive score conceived before ERP era, remains valid and reliable for predicting postoperative complications and death.

## Figures and Tables

**Table 1:** Implementation of our thoracic surgery enhanced recovery pathway.

Operative Phase	Elements Already in Place	What We Gave Up	Changes Induced by Our ERP
Pre	Preoperative assessment General patient information including smoking cessation	Stop food and drinks at midnight the day before surgery	Patient empowerment with dedicated information leaflet Carbohydrates intake
Per	Intraoperative warming Prophylactic antibiotics Goal directed fluid therapy Protective ventilation Targeted postoperative nausea prevention Regional anesthesia catheter	No use of non-steroid anti-inflammatory drugs use No strategy of chest tube management	Opiate analgesia avoidance strategy with non-steroid anti-inflammatory drugs use Single chest tube
Post	Venous thromboembolism prophylaxis Targeted postoperative nausea prevention Opioids for breakthrough pain Mobilization within 24 h	Pain evaluation at rest Continuous regional anesthesia Opioids personalized controlled anesthesia Maintenance of intravenous analgesics and fluids Feeding at POD 1 No consensus on chest tube management	Pain evaluation at rest and mobilization Personalized controlled regional anesthesia non-steroid anti-inflammatory drugs use Early infusion withdrawal Early feeding and mobilization at day 0 Consensus on chest tube management

**Table 2:** Population characteristics.

Features	First Period	Second Period
Total sample: (*n*)	166	1077
Age: mean (SD)	66 ± 10	66 ± 10
Gender (male): *n* (%)	86 (51.8)	616 (57.2)
Body mass index: mean (SD)	25 ± 4.7	25 ± 4.6
ASA Score: *n* (%)		
1	16 (9.6)	410 (38.0)
2	142 (85.5)	450 (41.8)
3	8 (4.8)	217 (20.2)
Smoking status: *n* (%)		
Current	45 (27.1)	331 (30.7)
Former	88 (53.0)	573 (53.2)
Never	33 (19.9)	166 (15.4)
Unknown		7 (0.7)
Surgical approach: *n* (%)		
Thoracotomy	149 (89.8)	903 (83.8)
Video-assisted thoracoscopy	11 (6.6)	144 (13.4)
Conversion **	6 (3.6)	30 (2.8)
Type of resection: *n* (%)		
Lobectomy	130 (78.3)	636 (59.1)
Bilobectomy		33 (3.1)
Pneumonectomy		59 (5.5)
Wedge resection		55 (5.1)
Segmentectomy	36 (21.7)	294 (27.2)
Lung cancer stage: *n* (%)		
Stage I	105 (63.3)	658 (61.1)
Stage II	41 (24.7)	211 (19.6)
Stage III	20 (12)	190 (17.6)
Comorbidities: n (%)		
Chronic obstructive pulmonary disease	59 (35.5)	419 (38.9)
Coronary artery disease	17 (10.2)	241 (22.4)
Pre-operative chemotherapy	11 (6.6)	73 (6.8)
Number of comorbidities: *n* (%)		
1	57 (34.3)	262 (24.3)
2	35 (21.1)	224 (20.8)
>3	21 (12.7)	591 (54.9)
Intermediate care unit hospitalization: *n* (%)	17 (10.2)	170 (15.8)

** Conversion were mainly due to unplanned pleural adhesions or anatomical abnormalities or scar tissue preventing safe vascular dissection.

**Table 3:** Patient outcomes.

Features	First Period	Second Period
Total postoperative adverse events: *n* (%)	50 (30)	326 (30.3)
Minor postoperative adverse events: *n* (%)	30 (18)	192 (17.8)
Major postoperative adverse events: *n* (%) **	20 (12)	134 (12.4)
Pneumonia	9	45
Atelectasis requiring bronchoscopy	2	5
Acute respiratory distress syndrome	0	14
Invasive mechanical ventilation > 24 h	1	20
Acute pulmonary edema	1	2
Pulmonary embolism	1	3
Myocardial infarction	0	2
Cardiac failure	0	1
Arhythmia	4	10
Neurological complications (stroke)	0	1
Acute renal failure	2	5
Deep vein thrombosis	0	1
Hemothorax	5	23
Chylothorax	1	2
Postoperative length of stay (day): median (IQR)	6 (5 to 8)	6 (5 to 9)
Patients back home after hospitalization: *n* (%)	139 (84)	991 (81)

** Some patients have had >1 postoperative events.

**Table 4:** Long term survival (first period): Univariable analysis. ASA: American Society of Anesthesiologists classification of physical status. COPD: Chronic Obstructive Pulmonary Disease. 95% CI: 95% Confidence Interval.

Features	*p*-Value	5-Year Survival Rate (95% CI)
Gender	0.32	
Men		73.3 (63.05–81.47)
Women		79.6 (69.36–87.07)
Age	0.0066	
≤70 years		80.3 (71.99–86.65)
>70 years		67.9 (54.52–78.91)
Histological type	0.026	
Carcinoid		78.2 (70.11–84.57)
Adenocarcinoma		68.2 (47.32–83.64)
Epidermoid carcinoma		91.7 (64.61–98.51)
Others		42.9 (15.82–74.95)
Mean tumoral diameter	0.057	
≤3 cm		80.3 (72.15–86.49)
>3 cm		66.9 (52.77–78.54)
Pleural invasion (*P*)	0.0026	
0		78.6 (69.9–85.32)
1		84.8 (69.08–93.35)
2		61.1 (38.62–79.7)
3		33.3 (9.68–70)
TNM Stage	0.049	
T1		84.3 (75.02–90.61)
T2		72.7 (59.77–82.72)
T3		59.1 (38.73–76.74)
T4		66.7 (30–90.32)
N0	0.000079	83.1 (75.9–88.46)
N1		46.2 (23.21–70.86)
N2		46.3 (25.35–68.69)
Lung cancer stage	0.00015	
I		86.7 (78.86–91.89)
II		63.4 (48.12–76.41)
III		50 (29.93–70.07)
BMI: (kg/m^2^)	0.60	
≤25		77.6 (67.56–85.17)
>25		75 (64.52–83.19)
ASA	0.9	
1		80 (54.81–92.95)
2		76.1 (68.4–82.34)
3		75 (40.93–92.85)
Resection type	0.52	
Segmentectomy		72.2 (56.01–84.15)
Lobectomy		77.5 (69.56–83.87)
Chronic obstructive pulmonary disease	0.061	
No		77.3 (68.47–84.29)
Yes		74.6 (62.2–83.94)
High blood pressure	0.36	
No		78.8 (69.23–86.02)
Yes		73.3 (62.37–82.02)
Coronary artery disease	0.53	
No		77 (69.55–83.05)
Yes		70.6 (46.87–86.72)
Lower limb atheroma	0.27	
No		75.1 (67.59–81.40)
Yes		87.5 (63.98–96.5)
Atrial fibrillation	0.96	
No		76.3 (68.98–82.27)
Yes		77.8 (45.26–93.68)
Diabetes	0.12	
No		78.1 (70.58–84.14)
Yes		65.2 (44.89–81.19)
Neoadjuvant treatment	0.26	
No		77.2 (69.98–83.17)
Yes		63.6 (35.38–84.83)
Smoking		
Never	0.43	80.8 (63.74–90.98)
Former		72.7 (62.55–80.90)
Current		80 8 (66.18–89.10)

**Table 5:** Long term survival (first period): Multivariable analysis.

Features	RR	IC	*p*
Pathological type			
Carcinoid	Ref		
Adenocarcinoma	1.75	1.09–2.81	0.32
Epidermoid carcinoma	3.06	1.18–7.92	
others	5.36	1.29–22.30	
Age			
22–70	Ref		
>70	2.50	1.31–4.78	0.0066
Pathological stage			
I	Ref		
II	2.28	1.56–3.32	
III	5.18	2.43–11.06	0.00015

**Table 6:** Impact of clinical and pathological variables on survival, follow-up and respiratory complications. ASA: American Society of Anesthesiologists classification of physical status. COPD: Chronic Obstructive Pulmonary Disease. Mean ± 95% CI: 95% Confidence Interval.

Features	Postoperative Death			Postoperative Complications				Respiratory Complications		
	Yes	No	*p*-Value	No	Minor	Major	*p*-Value	Yes	No	*p*-Value
Age, years	72.91 ± 4.98	66.59 ± 9.81	0.031	65.93 ±9.94	67.85 ± 9.89	69.00 ± 8.17	<0.001	69.36 ± 8.29	66.49 ± 9.86	0.0079
Gender: *n* (%)										
Men	10 (0.93)	606 (56.27)	0.049	367 (34.08)	61 (5.66)	33 (3.06)	<0.001	51 (4.73)	565 (52.46)	<0.001
Women	1 (0.09)	460 (42.71)		384 (35.65)	131 (12.16)	101 (9.39)		13 (1.21)	448 (41.60)	
BMI (kg/m^2^)	28.14 ± 4.88	25.24 ± 5.03	0.055	25.36 ± 5.05	25.20 ± 4.93	24.91 ± 5.04	0.64	25.72 ±5.68	25.24 ± 4.99	0.51
Smoking status: *n* (%)			0.94				0.011			0.0063
Current	3 (0.28)	328 (30.65)		225 (21.03)	61 (5.70)	45 (4.21)		24 (2.24)	307 (28.69)	
Former	6 (0.56)	567 (52.99)		386 (36.07)	107 (10.00)	80 (7.48)		39 (3.64)	534 (49.91)	
Never	2 (0.19)	164 (15.33)		134 (12.52)	23 (2.15)	9 (0.84)		1 (0.09)	165 (15.43)	
FEV1 (% of predicted)	68.27 ± 24.51	88.09 ± 20.52	0.0017	90.86 ± 19.73	81.70 ±21.16	80.33 ±21.08	<0.001	77.00 ± 19.23	88.58 ± 20.56	<0.001
FVC (% of predicted)	75.75 ± 11.67	97.39 ± 19.53	0.0020	99.71 ± 18.51	91.61 ± 20.94	90.21 ± 20.35	<0.001	87.93 ±19.94	97.69 ± 19.44	0.0026
DLCO (% of predicted)	61.22 ± 16.07	68.22 ± 18.05	0.16	69.96 ± 17.51	66.22 ± 17.78	61.41 ± 19.22	<0.001	61.41 ± 21.59	68.58 ± 17.69	0.031
Ischemic Heart Disease: *n* (%)			0.49				0.011			0.19
Yes	1 (0.09)	239 (22.21)		153 (14.22)	44 (4.09)	43 (4.00)		10 (0.93)	230 (21.38)	
no	10 (0.93)	826 (76.77)		597 (55.48)	148 (13.75)	91 (8.46)		54 (5.02)	782 (72.67)	
Chronic obstructive pulmonary disease: *n* (%)										
Yes	7 (0.66)	412 (38.25)	0.17	255 (23.68)	87 (8.08)	77 (7.15)	<0.001	38 (3.53)	381 (35.38)	<0.001
no	4 (0.37)	654 (60.72)		496 (46.05)	105 (9.75)	57 (5.29)		26 (2.41)	632 (58.68)	
Score ASA: *n* (%)										
I	1 (0.09)	409 (37.98)	0.048	309 (28.69)	57 (5.29)	44 (4.09)	<0.001	11 (1.02)	399 (37.05)	<0.001
II	5 (0.46)	445 (41.33)		323 (29.99)	82 (7.61)	45 (4.18)		22 (2.04)	428 (39.74)	
III	5 (0.46)	212 (19.68)		119 (11.05)	53 (4.92)	45 (4.18)		31 (2.88)	186 (17.27)	
Thoracoscore	4.59 ± 3.26	2.20 ± 1.84	0.0024	1.96 ± 1.42	2.71 ± 2.55	3.06 ± 2.49	<0.001	3.44 ± 2.87	2.16 ± 1.78	<0.001
Pneumonectomy: *n* (%)										
Yes	2 (0.19)	64 (5.94)	0.30	31 (2.88)	24 (2.23)	11 (1.02)	<0.001	3 (0.28)	63 (5.85)	0.82
No	9 (0.83)	1002 (93.04)		720 (66.85)	168 (15.60)	123 (11.42)		61 (5.66)	950 (88.21)	
Histology: (*n* = 1069): *n* (%)			0.094				<0.001			<0.001
Neuroendocrine	3 (0.28)	87 (8.14)		69 (6.45)	10 (0.94)	11 (1.03)		4 (0.37)	86 (8.05)	
Adenocarcinoma	5 (0.47)	709 (66.32)		521 (48.74)	119 (11.13)	74 (6.92)		31 (2.90)	683 (63.89)	
Epidermoid	3 (0.28)	207 (19.37)		119 (11.13)	50 (4.68)	41 (3.83)		26 (2.43)	184 (17.22)	
Others	-	55 (5.16)		37 (3.46)	10 (0.94)	8 (0.75)		3 (0.28)	52 (4.86)	
pT: *n* (%)			0.94				0.58			0.17
1 (a, b, c)	5 (0.46)	491 (45.59)		355 (32.96)	87 (8.08)	54 (5.01)		27 (2.50)	469 (43.55)	
2 (a, b)	4 (0.37)	326 (30.27)		231 (21.45)	57 (5.29)	42 (3.90)		15 (1.39)	315 (29.25)	
3	1 (0.09)	162 (15.04)		109 (10.12)	32 (2.97)	22 (2.04)		14 (1.30)	149 (13.84)	
4	1 (0.09)	87 (8.09)		56 (5.20)	16 (1.49)	16 (1.49)		8 (0.74)	80 (7.43)	

**Table 7:** Factors independently associated with the occurrence of complications. OR: Odd Ratio, CI: 95% Confidence Interval. The cut-off of 2.3 for Thoracoscore represents 75th percentile.

Features	Total Complications		*p*-Values
	OR	IC	
Age			
<70 years	Ref	1.04–1.93	*p* = 0.025
>70 years	1.42		
Gender			
Women	Ref	1.42–2.67	*p* = 0.00004
Men	1.95		
Thoracoscore			
<2.3	Ref	1.31–2.62	*p* = 0.00049
>2.3	1.85		
FEV1			
<80% predicted	Ref	0.46–0.86	*p* = 0.004
>80% predicted	0.63		
	**Major complications**		***p*-Values**
	OR	IC	
Gender			
Women	Ref	1.35–3.55	*p* = 0.0015
Men	2.19		
Thoracoscore			
<2.3	Ref	1.49–3.56	*p* = 0.00017
>2.3	2.30		
			*p* = 0.043
Smoking status			
- Never	Ref		
- Former	1.43	1.01–2.01	
- Current	2.03	1.02–4.05	
	**Respiratory complications**		***p*-Values**
	OR	IC	
Gender			
Women	Ref	1.32–6.39	*p* = 0.008
Men	2.90		
Thoracoscore			
<2.3	Ref	2.00–6.00	*p* = 0.000024
>2.3	3.63		
Smoking status			
- Never	Ref		*p* = 0.0052
- Former	2.12	1.25–3.58	
- Current	4.47	1.57–12.79	

## Data Availability

The data presented in this study are available on request from the corresponding author.

## Abbreviations and Acronyms

ERP	Enhanced recovery pathway
POD	postoperative day
PCRA	patient-controlled regional analgesia
